# Study on Roadheader Cutting Load at Different Properties of Coal and Rock

**DOI:** 10.1155/2013/624512

**Published:** 2013-11-04

**Authors:** Xueyi Li, Binbing Huang, Guoying Ma, Qingliang Zeng

**Affiliations:** College of Mechanical and Electronic Engineering, Shandong University of Science and Technology, Qingdao, Shandong 266590, China

## Abstract

The mechanism of cutting process of roadheader with cutting head was researched, and the influences of properties of coal and rock on cutting load were deeply analyzed. Aimed at the defects of traditional calculation method of cutting load on fully expressing the complex cutting process of cutting head, the method of finite element simulation was proposed to simulate the dynamic cutting process. Aimed at the characteristics of coal and rock which affect the cutting load, several simulations with different firmness coefficient were taken repeatedly, and the relationship between three-axis force and firmness coefficient was derived. A comparative analysis of cutting pick load between simulation results and theoretical formula was carried out, and a consistency was achieved. Then cutting process with a total cutting head was carried out on this basis. The results show that the simulation analysis not only provides a reliable guarantee for the accurate calculation of the cutting head load and improves the efficiency of the cutting head cutting test but also offers a basis for selection of cutting head with different geological conditions of coal or rock.

## 1. Introduction

There are various factors influencing the performance of roadheader in mechanized drivage operation, which mainly include the physical and mechanical properties of coal and rock and the structural parameters itself. As the direct working mechanism of roadheader, cutting head works in poor working conditions. The cutting picks wear and fracture and the fall-off of tool bit usually occur during the cutting process; in some driving working faces, there will be cutting hard degree that does not adapt to the cutting picks; all these phenomena result in the unreasonable use of cutting picks [1]. The performance of the cutting head directly determines the overall performance of the roadheader, and the improper selection of cutting picks with discomfort conditions of coal and rock will directly cause the failure of the cutting head and even the whole equipment, resulting in unnecessary accidents and losses.

Study on coal and rock breaking mostly concentrates on the coal and rock fragmentation effect and driving machinery itself. Chen et al. [2] studied the particle size distribution of detritus sampled from different excavation machines and methods. Song et al. [3] studied rock debris size and grain size distribution by the method of probability and statistics theory and the distribution rule of effect of rock properties and mechanical parameters was carried out. Li et al. [4, 5] researched the effect of rock fragmentation of static and dynamic loads through theoretical analysis and experimental studies. Wang [6] analyzed the stress, strain, and operating parameters on the deformation process of deformation and failure of coal and rock. Li [7], Guo et al. [8], and so on investigated the structure, load, and motion parameters of the cutting head itself. Copur et al. [9] found that the optimum cutting conditions of cutting ratio may be closely related to the uniaxial compressive strength and tensile strength of coal and rock through a comprehensive cutting test. Serhat [10] studied the driving machinery in the concrete construction of a coal mine in Turkey on the basis of the experimental research and found that the properties of the coal and rock have an important influence on cutting head instantaneous cutting rate and the cutting specific energy consumption. Bilgin et al. [11] studied some geological and geotechnical factors affecting the performance of a roadheader in an inclined tunnel. However, the domestic researchers have not researched the influence of the properties of coal and rock on roadheader cutting load with a systematic exposition, and foreign researchers mostly study the performance of cutting head by field test. But the field test is difficult to implement in our country due to the restriction of complex cutting head working environments as well as domestic coal mine geological conditions and other factors [12]. However, how to select the right type of driving machinery in different conditions of coal and rock excavation face in China's coal mining, urban underground construction, and other fields is the problem that needs to be solved urgently. Therefore, this paper aims at the influence of properties of coal and rock on roadheader cutting load; the cutting process of cutting head was studied by using dynamic simulation method, and the influence rule of characteristics of coal and rock was analyzed.

## 2. Coal and Rock Breaking Mechanism and Loads Analysis of Cutting Head

Since coal mine roadway construction or urban underground excavation and other needs of the construction, coal, and rock breaking become practical engineering challenges. At present, three categories of methods were applied which mainly contain mechanical breaking method, physical method, and chemical method, while the mechanical breaking method is the most widely used. This method is the use of mechanical stress which is applied on the coal and rock surface with special machine tools (such as cutting picks); the coal and rock load exceeds the limit and flakes from the surface. 

### 2.1. Coal and Rock Breaking Mechanism

The breaking process of cutting head can be divided into four stages as follows. (1) Stage of plastic deformation: the force of cutting pick on coal and rock increases gradually, leading the stress of coal and rock surrounding the contact to reach the yield limit firstly, resulting in plastic deformation. (2) Stage of cracks: when tensile stress suffered coal and rock exceeds its ultimate tensile strength with a further increase in the force with cutting picks, Hertz crack occurs. (3) Stage of dense nucleus formation: with the crack expanding, broken coal and rock powder move forward with the cutting picks, dense nucleus emerges, then a part of rock powder is ejected from the pick of blade surface, and dense nucleus volume decreases. (4) Stage of coal and rock breaking: with the further interaction of cutting picks and coal and rock, more rock powder becomes nucleus, and nucleus grows up; when the pressure exceeds a certain value, the coal and rock break, cutting load on picks decreases instantly, and a leapfrog cutting cycle is completed.

### 2.2. Mechanical Properties of Coal and Rock

It can be seen that the properties of coal and rock have an important effect on the cutting process from coal and rock breaking mechanism. The properties will affect the load, cutting efficiency, and so on. Specifically, the hardness of coal and rock reflects the ability to resist external force in the local area so that it will not be destroyed; the strength of coal and rock, including compressive strength, tensile strength, and shear strength, is the limiting stress when the coal and rock start to be broken by external force, which is often used as the leading indicator of evaluating the bearing capacity; the elasticity, plasticity, and brittleness of coal and rock also reflect the relationship between load and deformation of coal and rock breaking; the firmness coefficient of coal and rock is a reflection of the ability to resist external fragmentation as a comprehensive index; the firmness coefficient of coal and rock (protodyakonov scale of hardness) *f* and uniaxial compressive strength of coal and rock limit *f*_*c*_ (MPa) satisfy the relation in

(1) $$\begin{array}{c} f=\frac{f_{c}}{10}. \end{array}$$

Research has shown that the firmness coefficient of coal and rock is the most reliable indicator to characterize the properties of coal and rock during the cutting process, which is widely used in the mining and exploration industries [13]. The current domestic machine production enterprises also mostly employ the coefficient as the standard for formulating the technical indexes of the product nowadays.

### 2.3. Loads Analysis of Cutting Head

The loads cutting head suffers affecting the performance and service life directly, which is often used as an evaluation basis for cutting head performance. However, the loads on cutting head and picks are nonlinear due to the nonlinear contact of coal and rock, nonlinear material, and the multiple arrangements of cutting picks. These lead to no accepted, precise calculation method of analyzing the loads on cutting head and picks.

(1)  *Load Calculation of Cutting Picks.* We often use the formula given by the former Soviet Union scholar to calculate the loads of cutting picks as follows [1]:

(2) $$\begin{array}{c} P_{zi}=P_{k}\left[k_{T}k_{g}k_{y}^{\prime }\left(0.25+0.18th\right)+0.1S_{j}\right],\\ P_{yi}=P_{zi}\left(0.15+0.00056P_{k}\right)\frac{2.5}{h^{0.4}},\\ P_{xi}=P_{zi}\left[\frac{C_{1}}{\left(C_{2}+h\right)}+C_{3}\right]\frac{h}{t}, \end{array}$$

where *P*_*zi*_, *P*_*yi*_, and *P*_*xi*_ are cutting resistance, tractive resistance, and side resistance on cutting pick numbered *i*, respectively (N); *P*_*k*_ is the contact strength (MPa); *k*_*T*_ is the coefficient of cutting pick type; *k*_*g*_ is the coefficient of cutting pick geometrical shape; *k*_*y*_′ is the coefficient of head shape of cutting pick; *t* is the average transversal spacing (mm); *h* is the average cutting thickness (mm); *S*_*j*_ is the projected area of cutting pick after blade surface in the traction direction (mm^2^); *C*_1_, *C*_2_, and *C*_3_ are chip influence coefficient. The value of parameter in the formula can be selected by [1] except *P*_*k*_.

The contact strength *P*_*k*_ and the protodyakonov scale of hardness *f* have certain corresponding relations as shown in Table 1.

**Figure 1:**
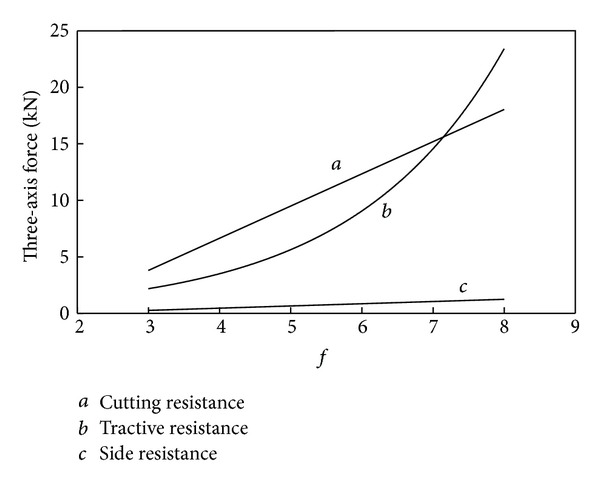
Relationship between three-axis force and *f*.

The curve of relationship between three-axis force and *f* can be drawn according to corresponding relationships in formula (2) and Table 1, which is shown in Figure 1.

The change trend between three-axis force and the firmness coefficient of coal and rock can be seen from Figure 1, which reflects the change law of loads on cutting picks with the properties of coal and rock.

(2)  *Load Calculation of Cutting Head.* The theoretical value of loads on cutting head can be obtained by combining the loads on cutting picks that participate in cutting at any time based on load calculation of single cutting pick.

**Figure 2:**
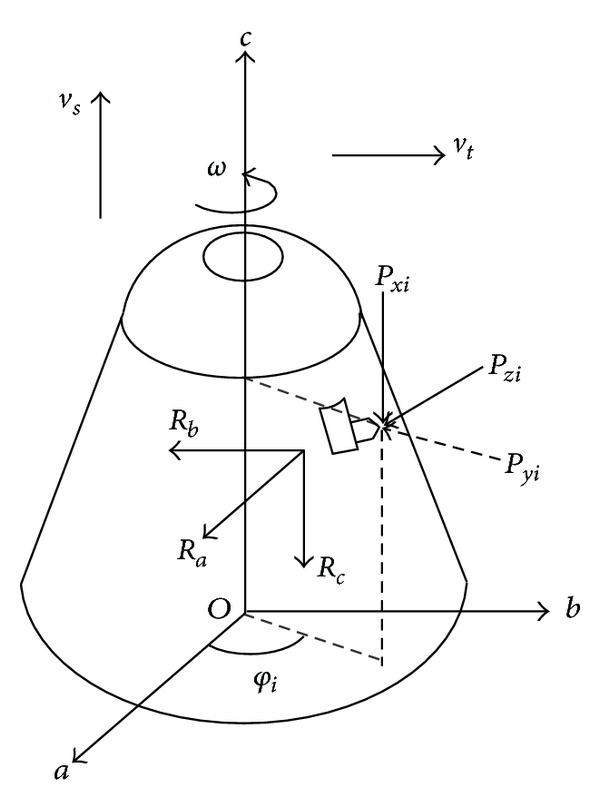
Load diagram of cutting head.

Define the central shaft of gyration of cutting head as *c* axis and set up a coordinate system *Oabc* as shown in Figure 2. Establish the simplified load diagram of the cutting head for convenience due to the actual cutting head model being complex, in which, *P*_*zi*_, *P*_*yi*_, and *P*_*xi*_ are loads on single cutting pick, and the value of them can be calculated according to formula (2); *ω* is the direction of rotation of the cutting head; *v*_*s*_ is the drilling direction of cutting head; *v*_*t*_ is the swing direction. The three-axis force of cutting head can be obtained by the following formula:

(3) $$\begin{array}{c} R_{a}=\overset{n}{\underset{i=1}{\sum }}\left({P_{z}}_{i}\cos\varphi_{i}+{P_{y}}_{i}\sin\varphi_{i}\right),\\ R_{b}=\overset{n}{\underset{i=1}{\sum }}\left({P_{z}}_{i}\sin\varphi_{i}-{P_{y}}_{i}\cos\varphi_{i}\right),\\ R_{c}=\overset{n}{\underset{i=1}{\sum }}{P_{x}}_{i}, \end{array}$$

where *R*_*a*_, *R*_*b*_, and *R*_*c*_ are the resultant force of cutting head along three coordinate axes; *n* is the number of picks which participate in cutting at this moment; *φ*_*i*_ is the position angle of a single cutting pick. 

It can be seen from formulae (2) and (3) that the properties of coal and rock have an effect on loads of both single cutting pick and whole cutting head. However, the loads are dynamic and the process of cutting is complex; the equation established on theoretical analysis cannot describe the complex process of coal and rock cutting comprehensively and accurately. The impact of the transient load causes the static strength of the cutting head damage; the dynamic response of the load will cause the vibration of the cutting head which result in fatigue failure. Therefore, this paper analyzes the dynamic cutting process by finite element simulation method based on the traditional theory to get the dynamic loads with different properties of coal and rock.

## 3. Finite Element Simulation of Cutting Process

### 3.1. Definition of Material Parameter

In order to obtain reliable results of finite element simulation analysis, the definition of material is of great importance. However, some of the material parameters are difficult to obtain in practice in the analysis; as mentioned above, the firmness coefficient of coal and rock is the indicator to characterize the properties of coal and rock, which can derive the main mechanical parameters required for analysis.

The uniaxial compressive strength of coal and rock limit *f*_*c*_ and firmness coefficient of coal and rock *f* satisfy the relation according to formula (1):

(4) $$\begin{array}{c} f_{c}=10f. \end{array}$$

The relationship between elasticity modulus of coal and rock *E*_*c*_ and uniaxial compressive strength *f*_*c*_ is [14]

(5) $$\begin{array}{c} E_{c}=0.043\rho_{0}^{1.5}\sqrt{f_{c}}, \end{array}$$

where *ρ*_0_ is the density of coal and rock before breaking.

The shear elasticity *G* and elasticity modulus *E*_*c*_ of coal and rock satisfy the relation as follows according to elastic theory:

(6) $$\begin{array}{c} G=\frac{E_{c}}{2\left(1+\upsilon \right)}, \end{array}$$

where *υ* is the Poisson ratio of the material.

The relationship between shear elasticity *G* and firmness coefficient of coal and rock *f* can be deduced by formulae (4), (5), and (6) as follows:

(7) $$\begin{array}{c} G=0.043\rho_{0}^{1.5}\frac{\sqrt{10f}}{2\left(1+\upsilon \right)}. \end{array}$$

Define the cutting head as a rigid body after an overall consideration of simulation time and the mechanical behavior during cutting contact of the cutting head. 

### 3.2. Finite Element Modeling

Table 2 shows the structure parameter of cutting head. Build the parametric entity model of cutting head for cutting simulation using the APDL language in ANSYS system due to the diversity and complexity of cutting head's structure, which can greatly improve the efficiency of analysis.

**Figure 3:**
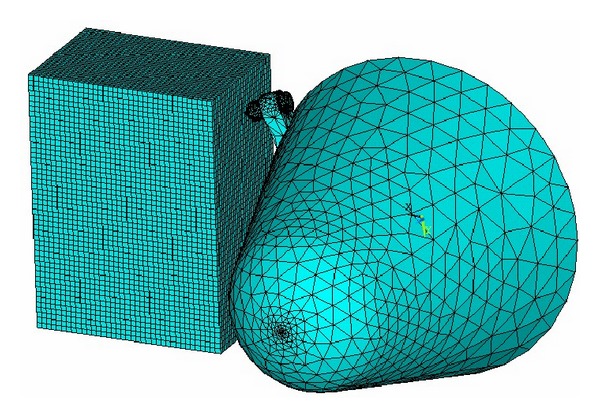
FE model of cutting process with a single pick.

**Figure 4:**
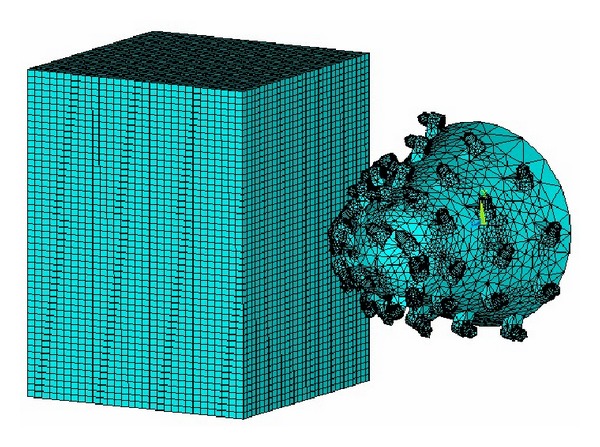
FE model of cutting process with cutting head.

Select SOLID 164 unit for analysis and mesh the cutting head and coal and rock with reasonable manner; then define the PART and contact type. In order to analyze the dynamic characteristic of single cutting pick and entire cutting head, establish two models, respectively, as shown in Figures 3 and 4. 

### 3.3. Loading, Constraint, and Solving

Apply the rotation velocity with 36 r/min and horizontal swing velocity with 1.5 m/min to the cutting head after finite element modeling. Add nonreflect boundary conditions to surfaces of coal and rock without cutting in the simulation system and limit the degrees of freedom of other directions. Add output contact surface reaction keyword “RCFORC” for the force output of cutting head.

Set the solving time according to the rotation and horizontal swing velocity of the cutting head. Select a reasonable quality scaling factor and time step scale factor. Submit to solve after completion of all operations.

In order to analyze the cutting load rule at different properties of coal and rock, simulate the cutting process with firmness coefficient of different coal and rock from 3 to 8; the load characteristics of cutting pick and cutting head were obtained.

## 4. Influence of Properties of Coal and Rock on Roadheader Cutting Load

It can be seen that the properties of coal and rock have an effect on cutting load of both cutting pick and cutting head according to coal and rock breaking mechanism and calculation formula of cutting head. 

### 4.1. Influence of Properties of Coal and Rock on Cutting Pick Load

**Figure 5:**
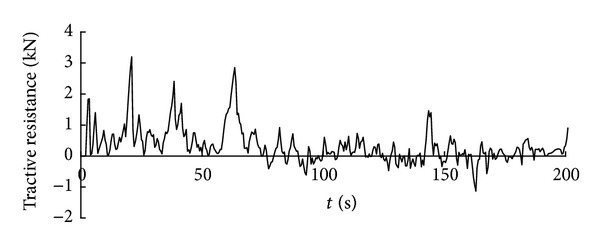
Time history curve of tractive resistance on cutting pick.

**Figure 6:**
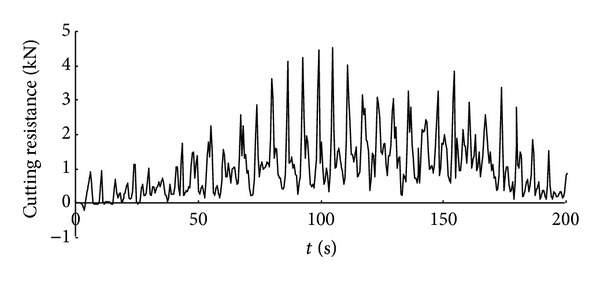
Time history curve of cutting resistance on cutting pick.

**Figure 7:**
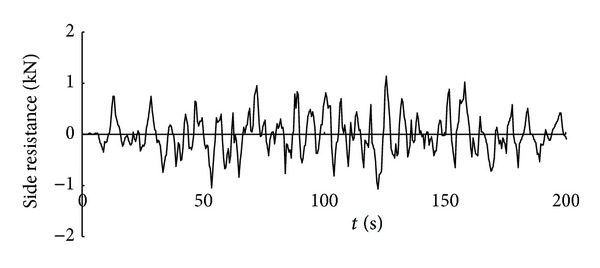
Time history curve of side resistance on cutting pick.

**Figure 8:**
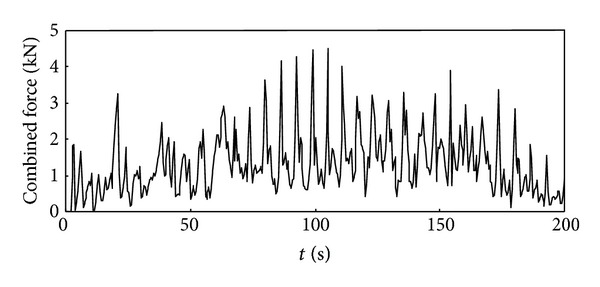
Time history curve of the combined force on cutting pick.

Time history curve of the three-axis force and combined force on cutting pick are obtained through simulation analysis of cutting process with cutting pick when the firmness coefficient of coal and rock is 3, which is shown in Figures 5, 6, 7, and 8.

It can be seen from the above curves that the cutting resistance, traction resistance, side resistance, and combined force showed irregular fluctuations, which relate to the anisotropy of the material and the break of coal and rock. The phenomenon is consistent with the coal and rock breaking mechanism, the force increases before coal and rock break, and cutting load on picks decreases instantly after the break, and a leapfrog cutting cycle is completed. 

In order to obtain change rule of the three-axis force on cutting pick, count the maximum and the average value of three-axis force when the firmness coefficient of coal and rock changes from 3 to 8; the load statistics of cutting process with a single pick is shown in Table 3.

**Figure 9:**
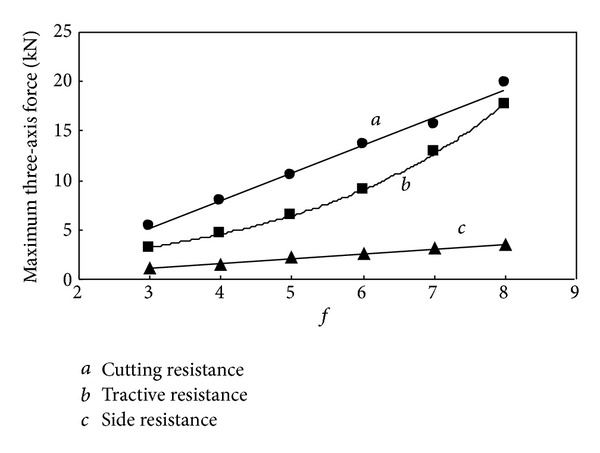
Relationship between maximum three-axis force and *f*.

**Figure 10:**
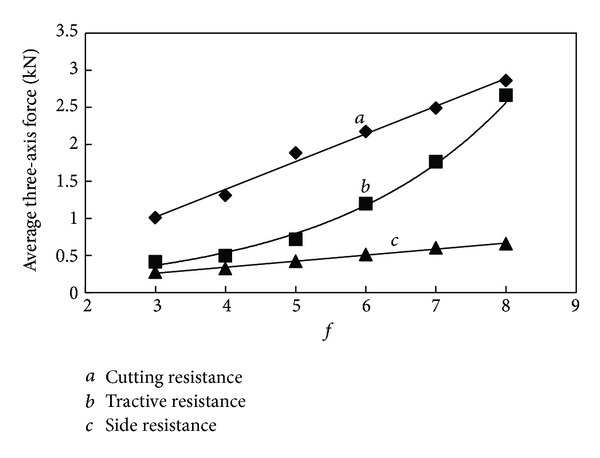
Relationship between average three-axis force and *f*.

It can be seen in the statistical data from Table 3 that the maximum and average cutting resistance and traction resistance are much larger than the side resistance. The value of three-axis force increases along with the increase of the firmness coefficient. In order to provide a better understanding of the relationship between them, draw the relation curve between three-axis force and *f* shown in Figures 9 and 10.

From the relation curve it can be seen that the cutting resistance and side resistance are in linear correlation with the firmness coefficient; however the traction resistance increases along with the firmness coefficient in exponential form. The variation tendency agreed with computational formula (2); however, in value, the maximum force is slightly greater than three theoretical values, and the average force is much smaller than the theoretical value, and the correlation between average three-axis force and firmness coefficient is worse than maximum ones, which indicate that the theoretical formula tends to calculate the maximum load.

The results can be seen from the comparison; the numerical simulation can accurately obtain the cutting load of cutting picks during the cutting process. On this basis, study of the load of the cutting head provided more practical significance.

### 4.2. Influence of Properties of Coal and Rock on Cutting Head Load

**Figure 11:**
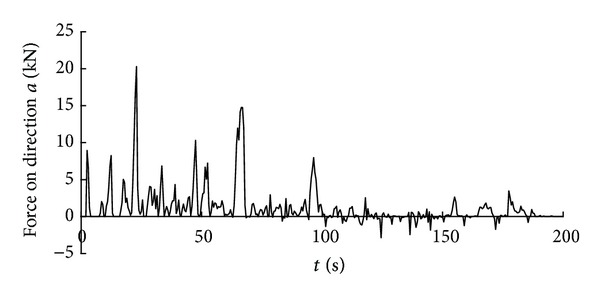
Time history curve of the force on direction *a* on cutting head.

**Figure 12:**
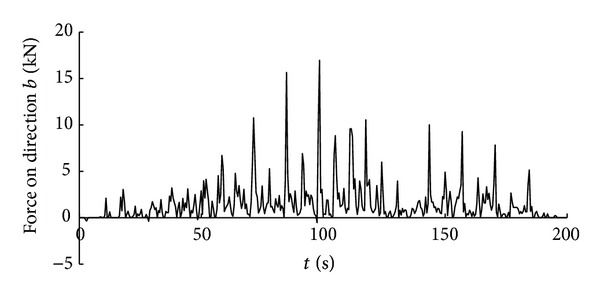
Time history curve of the force on direction *b* on cutting head.

**Figure 13:**
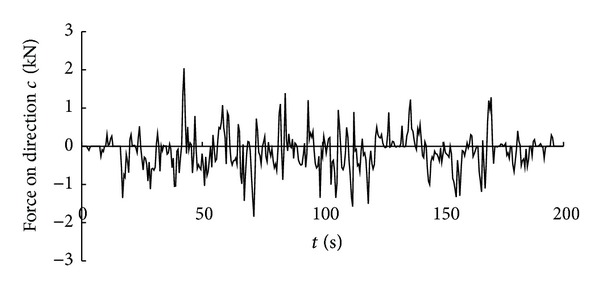
Time history curve of the force on direction *c* on cutting head.

**Figure 14:**
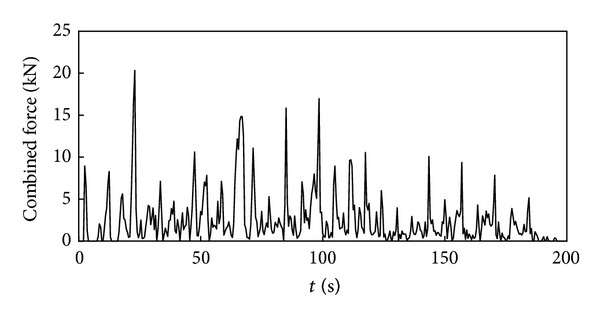
Time history curve of the combined force on cutting head.

In order to get the time history curve of the three-axis force and combined force on the entire cutting head, make the simulation of cutting process using the same method on the basis of single cutting pick cutting simulation. The curves are shown in Figures 11, 12, 13, and 14.

Similar to the forces on cutting pick, the forces that the entire cutting head suffered also showed irregular fluctuations, so the value of the forces is changing and the load that cutting head suffered is dynamic load. As a result, the traditional theoretical analysis cannot accurately calculate the load characteristics of the cutting head and in order to obtain the dynamic loads of cutting head during cutting process can only through FEM analysis. 

**Figure 15:**
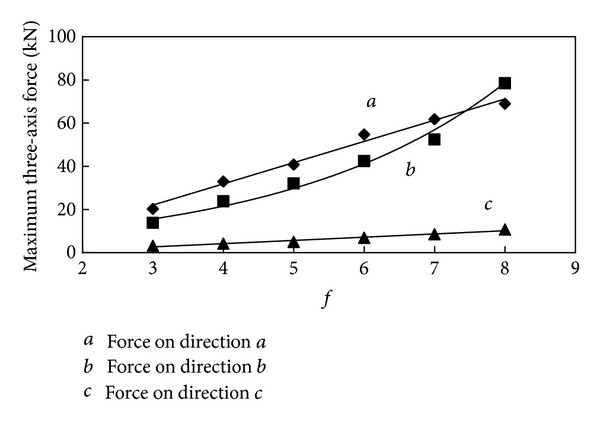
Relationship between maximum three-axis force and *f*.

**Figure 16:**
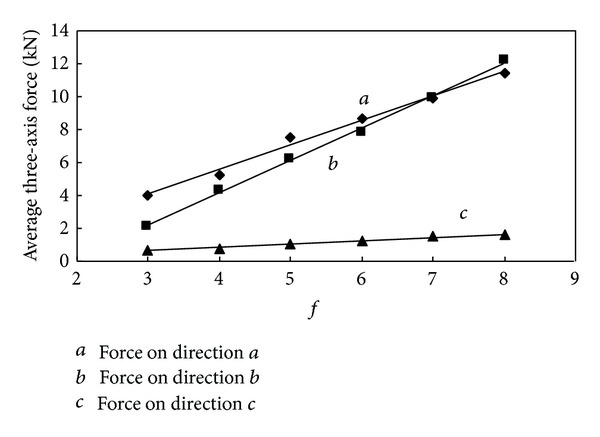
Relationship between average three-axis force and *f*.

In order to obtain change rule of the three-axis force on cutting head as well, draw the statistics table of cutting process with cutting head and relation curve between three-axis force and *f*, which are shown in Table 4 and Figures 15 and 16. 

We can see clearly from the relation curve that the maximum three-axis force of cutting head changes in the same way as the cutting pick, but the correlation is not as good as the force of cutting pick. The average three-axis force of cutting head increases linearly along with the increase of the firmness coefficient. As can be seen from the results of the analysis, the difficulty of cutting increases along with properties of coal and rock enhancements; cutting head load increases significantly, but the three-axis force increases at different rates. When the firmness coefficient of coal and rock increases to a certain extent, traction resistance of cutting head becomes the largest one and the side resistance is always at a low level. From the simulation analysis, variation trend of the cutting head loads with the firmness coefficient can be seen clearly, but making the appropriate test is a reasonable choice of roadheader cutting head for different geological conditions of coal and rock.

## 5. Conclusions

The poor working environment of roadheader cutting head determines the limitations of field testing, and laboratory test model cannot accurately express the complexity of the actual job site conditions. Roadheader cutting head load has an important influence on the machine's performance, as the object of cutting and coal and rock characteristics have a significant impact on the cutting load. Traditional methods cannot accurately obtain the theoretical dynamic loads of cutting process. This paper uses the method of numerical simulation of cutting process of cutting pick and cutting head and gets the cutting load rule at different properties of coal and rock. Numerical simulation method can take an integrated consideration of the characteristics of coal and rock to the impact of the cutting process, which greatly improves the cutting test results.

There are many parameters that can influence the cutting load; taking an overall consideration of all the parameters is not feasible. However, taking the cutting simulation with appropriate coal and rock parameters for different coal and rock conditions can provide the basis for selection of the head for different geological conditions.

## Figures and Tables

**Table 1:** Corresponding relationship between *P*_*k*_ and *f*.

*f*	3	4	5	6	7	8
*P*_*k*_	230	350	490	650	800	1000

**Table 2:** Structure parameter of cutting head.

Length (mm)	Diameter (mm)	Cone angle (°)	Helical blade head number	Cutting angle (°)	Inclination angle (°)
900	*ϕ* 850	35	3	45	8

**Table 3:** Load statistics of cutting process with a single pick.

Three-axis force (N)	*f* = 3		*f* = 4		*f* = 5		*f* = 6		*f* = 7		*f* = 8	
	Maximum	Average	Maximum	Average	Maximum	Average	Maximum	Average	Maximum	Average	Maximum	Average
Cutting resistance	5392	1009	7930	1310	10499	1880	13677	2169	15642	2487	19765	2860

Traction resistance	3203	413	4578	495	6417	714	8935	1198	12840	1764	17643	2660

Side resistance	1150	275	1518	319	2272	420	2620	512	3124	601	3467	655

**Table 4:** Load statistics of cutting process with cutting head.

Three-axis force (N)	*f* = 3		*f* = 4		*f* = 5		*f* = 6		*f* = 7		*f* = 8	
	Maximum	Average	Maximum	Average	Maximum	Average	Maximum	Average	Maximum	Average	Maximum	Average
Force on direction *a*	20290	4036	31720	5241	41996	7524	54708	8676	62568	9948	79060	11439

Force on direction *b*	16594	2065	22890	4312	32085	6224	44675	7847	64200	9872	88215	12229

Force on direction *c*	2043	687	3036	798	4544	1050	5240	1279	6248	1502	6934	1637
